# Robust Association Tests for the Replication of Genome-Wide Association Studies

**DOI:** 10.1155/2015/461593

**Published:** 2015-08-04

**Authors:** Jungnam Joo, Ju-Hyun Park, Bora Lee, Boram Park, Sohee Kim, Kyong-Ah Yoon, Jin Soo Lee, Nancy L. Geller

**Affiliations:** ^1^Biometric Research Branch, Research Institute and Hospital, National Cancer Center, Gyeonggi-do, Goyang-si 410-769, Republic of Korea; ^2^Department of Statistics, Dongguk University, Seoul 100-715, Republic of Korea; ^3^Lung Cancer Branch, Research Institute and Hospital, National Cancer Center, Gyeonggi-do, Goyang-si 410-769, Republic of Korea; ^4^Office of Biostatistics Research, National Heart, Lung and Blood Institute, Bethesda, MD 20892-7938, USA

## Abstract

In genome-wide association study (GWAS), robust genetic association tests such as maximum of three CATTs (MAX3), each corresponding to recessive, additive, and dominant genetic models, the minimum *p* value of Pearson's Chi-square test with 2 degrees of freedom, and CATT based on additive genetic model (MIN2), genetic model selection (GMS), and genetic model exclusion (GME) methods have been shown to provide better power performance under wide range of underlying genetic models. In this paper, we demonstrate how these robust tests can be applied to the replication study of GWAS and how the overall statistical significance can be evaluated using the combined test formed by *p* values of the discovery and replication studies.

## 1. Introduction

With the advance of biotechnology and substantial reduction of genotyping costs, a genome-wide association study (GWAS) using hundred thousand markers in several thousand individuals is now increasingly utilized and has been successful in detecting genetic associations across the entire genome with complex human traits [1–6]. Among many challenges this application holds; development of more efficient and robust statistical methodologies with higher power to detect an association with a single marker has been one of the most important statistical issues, given that effects of individual markers are usually characterized as being small to moderate. One attempt to overcome this challenge is focused on developing efficient tests that are robust against underlying genetic model misspecification.

Two most frequently used association tests are the allele-based test (ABT) and the genotype-based test (GBT). ABT compares the allele frequencies between cases and controls, while GBT compares the genotype distributions of cases and controls. The Cochran-Armitage trend test (CATT) [7, 8] is a popular GBT which takes into account the underlying genetic model. It is well known, however, that the ABT may inflate type I error when Hardy-Weinberg equilibrium (HWE) does not hold in the samples [9]. Even under HWE, when the genetic model is recessive or dominant, the ABT may suffer from serious power loss. On the other hand, the CATT does not depend on HWE, but to apply the CATT the choice of scores optimal for the underlying genetic model needs to be specified. For complex diseases, the genetic model is usually unknown and robust tests such as the maximum of three CATTs (MAX3) [10] and the maximum efficiency robust test (MERT) [11, 12] are preferable. Alternatively, Zheng and Ng [13] and Joo et al. [14] proposed a two-phase analysis based on the genetic model selection (GMS) and genetic model exclusion (GME). Moreover, an alternative approach was proposed by the Wellcome trust case-control consortium (WTCCC) [5] which used a minimum *p* value of Pearson's Chi-square test and additive CATT, and the asymptotic properties of this approach were studied in detail by Joo et al. [15]. These methods provide better or comparable power performance than some of the robust tests such as MAX3.

In this paper, we illustrate how these robust tests can be applied to a replication study of GWAS and how overall statistical significance can be evaluated using the combined test formed by *p* values of the discovery and replication studies. The importance of replication or validation in GWAS has been well recognized [16, 17], and joint analysis in a two-stage design of GWAS has been proved to be more powerful than replication-based analysis and has been widely conducted in GWAS with a variety of phenotypes of interest [18, 19].

The paper is organized as follows. We first describe the data structures and notation and review existing robust association tests for a single data set. Then we describe how to obtain the *p* value for the replication data set, given the significant result of the discovery stage, using robust tests. In the next section, a combined test of the *p* values of the discovery and replication data sets is proposed, together with the way to evaluate the statistical significance for the combined test. Simulation studies are conducted to compare the type I error rates and powers of various analytical strategies. For illustration purposes, the summarized methods are applied to a non-small-cell lung cancer data set and at the end there is a discussion.

## 2. Methods

### 2.1. Data and Notation

For a marker with two alleles *A* and *B*, let the frequencies of *B* in cases and controls be *p* = *P*(*B*∣case) and *q* = *P*(*B*∣control). Denote three genotypes by *G*_0_ = *AA*, *G*_1_ = *AB*, and *G*_2_ = *BB*. In case-control association studies, *r* cases and *s* controls are independently sampled from each population. The observed genotype counts for (*G*_0_, *G*_1_, *G*_2_) are (*r*_0_, *r*_1_, *r*_2_) in the cases and (*s*_0_, *s*_1_, *s*_2_) in the controls. Disease prevalence is denoted by *k* = *P*(disease) and penetrance by *f*_*i*_ = *P*(disease∣*G*_*i*_) for *i* = 0,1, 2. Two genotype relative risks (GRRs) are denoted by *λ*_1_ = *f*_1_/*f*_0_ and *λ*_2_ = *f*_2_/*f*_0_ using *f*_0_ > 0 as baseline penetrance. Under the null hypothesis of no association *H*_0_ : *f*_0_ = *f*_1_ = *f*_2_ = *k* or alternatively *H*_0_ : *λ*_2_ = *λ*_1_ = 1. Genetic model is recessive (REC), additive (ADD), multiplicative (MUL), and dominant (DOM) when *λ*_1_ = 1, *λ*_1_ = (1 + *λ*_2_)/2, *λ*_1_ = *λ*_2_^1/2^, and *λ*_2_ = *λ*_1_, respectively.

### 2.2. Review of Association Tests for a Single Data Set

The association in case-control studies can be tested using various methods which have been extensively studied. The general association between the disease status and the SNP can be tested using Pearson's Chi-square test which has an asymptotic Chi-square distribution with 2 degrees of freedom under *H*_0_. The test is given by

(1) $$\begin{array}{c} T_{\text{chi}2}=\overset{2}{\underset{j=0}{\sum }}\frac{{\left(r_{j}-n_{j}r/n\right)}^{2}}{n_{j}r/n}+\overset{2}{\underset{j=0}{\sum }}\frac{{\left(s_{j}-n_{j}s/n\right)}^{2}}{n_{j}s/n}, \end{array}$$

where *n*_*i*_ = *r*_*i*_ + *s*_*i*_ for *i* = 0,1, 2 and *n* = *r* + *s*. Under Hardy-Weinberg equilibrium (HWE), an allele-based test (ABT) and CATT with scores (0, *x*, 1) for (*G*_0_, *G*_1_, *G*_2_), where 0 ≤ *x* ≤ 1, are given by

(2) $$\begin{array}{c} Z_{\text{ABT}}=\frac{n^{1/2}\left\{2r\left(2s_{0}+s_{1}\right)-2s\left(2r_{0}+r_{1}\right)\right\}}{{\left\{2rs\left(2n_{0}+n_{1}\right)\left(n_{1}+2n_{2}\right)\right\}}^{1/2}},\\ Z_{x}=\frac{n^{1/2}\sum_{i=0}^{2}x_{i}\left(sr_{i}-rs_{i}\right)}{{\left[rsn\left\{n\sum_{i=0}^{2}x_{i}^{2}n_{i}-{\left(\sum_{i=0}^{2}x_{i}n_{i}\right)}^{2}\right\}\right]}^{1/2}}, \end{array}$$

where (*x*_0_, *x*_1_, *x*_2_) = (0, *x*, 1) [9]. The optimal choices of *x* for the recessive (REC), additive/multiplicative (ADD/MUL), and dominant (DOM) models are *x* = 0,1/2 and 1, respectively [9, 20]. Both *Z*_*x*_ and *Z*_ABT_ asymptotically follow a standard normal distribution under *H*_0_. *Z*_*x*_ can be used even when HWE does not hold. However, without the HWE assumption, *Z*_ABT_ does not follow a standard normal distribution due to the correlation between two alleles.

A robust test, MAX3 proposed by Friedlin et al. [10], can be obtained by taking the maximum of three CATTs under the three genetic models as MAX3 = max⁡(|*Z*_0_ | , |*Z*_1/2_ | , |*Z*_1_|). Parametric bootstrap or permutation methods can be used to find the *p* value of MAX3 [4].

Let the *p* values of Pearson's Chi-square test and CATT under the additive genetic model *Z*_1/2_ be *P*_chi2_ and *P*_1/2_, respectively. WTCCC [5] proposed an alternative robust test MIN2 = min⁡(*P*_chi2_, *P*_1/2_). Joo et al. [15] derived the asymptotic null distribution of MIN2 and using their result the *p* value of MIN2 can be obtained as

(3) PMIN2=12exp⁡−12H1−1(1−MIN2)+12MIN2 −12π∫H1−11−MIN2−2log⁡⁡MIN2e−v/2arcsin2H1−11−MIN2v−1dv,

where *H*_1_ and *H*_2_ are the cumulative distributions of Chi-square distributions with 1 and 2 degrees of freedom.

On the other hand, Song and Elston [21] considered a Hardy-Weinberg disequilibrium trend test (HWDTT) given by

(4) $$\begin{array}{c} Z_{H}=\frac{{\left(rs/n\right)}^{1/2}\left({\hat{\Delta }}_{p}-{\hat{\Delta }}_{q}\right)}{\left\{1-n_{2}/n-n_{1}/\left(2n\right)\right\}\left\{n_{2}/n+n_{1}/\left(2n\right)\right\}}, \end{array}$$

where ${\hat{\Delta }}_{p}={\hat{p}}_{2}-{\left({\hat{p}}_{2}+{\hat{p}}_{1}/2\right)}^{2}$ and ${\hat{\Delta }}_{q}={\hat{q}}_{2}-{\left({\hat{q}}_{2}+{\hat{q}}_{1}/2\right)}^{2}$ are the estimates of Δ_*p*_ and Δ_*q*_, where ${\hat{p}}_{i}=r_{i}/r$ and ${\hat{q}}_{i}=s_{i}/s$. Here, Δ denotes the Hardy-Weinberg disequilibrium (HWD) coefficient defined by *Pr*(*BB*)−{*Pr*(*AB*)/2 + *Pr*(*BB*)}^2^ and Δ_*p*_ and Δ_*q*_ denote the HWD coefficient in cases and controls, respectively.

Zheng and Ng [13] used the information contained in the signs of (Δ_*p*_, Δ_*q*_) to determine the genetic models in their two-phase method. Their two-phase statistic *Z*_GMS_ is given by *Z*_GMS_ = *Z*_0_ if *Z*_*H*_ > *c*, *Z*_1_ if *Z*_*H*_ < −*c*, and *Z*_1/2_ otherwise, where *c* = Φ^−1^(1 − *α*_*H*_) for *α*_*H*_ = 0.05. The asymptotic correlations between *Z*_*H*_ and three CATTs under HWE were derived and the significance level was adjusted accordingly to control the desired type I error. Based on the observation that this method assumes *B* is the risk allele, Joo et al. [14] studied the behavior of *Z*_GMS_ when either one of the alleles can be a risk allele. They chose the risk allele based on the sign of *Z*_1/2_; that is, if *Z*_1/2_ > 0, *B* is the risk allele, and *Z*_0_, *Z*_1/2_, and *Z*_1_ are chosen for REC, ADD, and DOM models, respectively. If *Z*_1/2_ < 0, the respective test statistics are chosen to be −*Z*_1_, −*Z*_1/2_, and −*Z*_0_. They incorporate this property in defining the test statistic for genetic model selection (*Z*_GMS_) and calculating the *p* value. Let Θ_0_(*z*) = {*z* : *z* > *c*}, Θ_1/2_(*z*) = {*z* : |*z* | <*c*}, and Θ_1_(*z*) = {*z* : *z* < −*c*}. Then, the *p* value of this method can be obtained by

(5) pGMS=2∑x=01∫Θx(zH)∫0∞∫t∞ϕx(zx,z1/2,zH)dzxdz1/2dzH +2∫Θ1/2(z)Φ(−t∧0)+ρ1/2z1−ρ1/221/2dΦ(z),

where *ρ*_*x*_ = Corr(*Z*_*x*_, *Z*_*H*_) in (5) and *ρ*_*x*,1/2_ = Corr(*Z*_*x*_, *Z*_1/2_) (*x* = 0,1) are replaced by their consistent estimates. Here, *t* = *z*_GMS_ and (−*t*∧0) = min⁡(−*t*, 0). Moreover, *z*_GMS_ and *z*_1/2_ are the observed values of *Z*_GMS_ and *Z*_1/2_, respectively.

While studying the properties of GMS, Joo et al. [14] noticed that the probability of selecting the true recessive or dominant models using *Z*_*H*_ is very low especially for low to moderate GRRs, but the unlikely genetic model can be successfully excluded. This led to genetic model exclusion method *Z*_GME_ which is the same as the *Z*_GMS_ described above except *Z*_*x*_ for *x* = 0,1/2,1 is replaced by *Z*_x_^*^ where $Z_{x}^{*}=(Z_{x}+Z_{1/2})/{\left\{2(1+{\hat{\rho }}_{x,1/2})\right\}}^{1/2}$. And the *p* value of GME can be obtained as

(6) pGME=2∑x=01∫ΘxzH∫0∞∫L∞ϕxzx,z1/2,zHdzxdz1/2dzH +2∫Θ1/2(z)Φ(−t∧0)+ρ1/2z1−ρ1/221/2dΦ(z),

where $L=t{\left\{2(1+{\hat{\rho }}_{x,1/2})\right\}}^{1/2}-z_{1/2}$ for *t* = *z*_GME_.

### 2.3. *p* Value of Replication Data Using the Robust Method

In the discovery stage, the *p* value of robust association tests, including MAX3, MIN2, *Z*_GMS_, and *Z*_GME_, can be obtained as described in Section 2.2. For the *p* value of replication data using the robust method, we use the same analytic method that was used for discovery and the risk allele identified by it [16]. This means that when the best test statistic or genetic model is selected in the discovery stage, the replication stage will adopt the discovery stage selection and the direction of association.

Suppose that, for simplicity of notation, our interest is in GWAS with two stages, one for discovery and the other for replication, although the methodology described below can be extended to multistages for replication. Let *Z*_*x*_^(*i*)^ for *x* = 0,1/2,1 be the CATT optimal for recessive, additive, and dominant models and let *P*_*x*_^(*i*)^ be corresponding *p* value for *i*th stage (*i* = 1 for discovery and *i* = 2 for replication stages). Also, denote $Z_{x}^{(i)*}=(Z_{x}^{\left(i\right)}+Z_{1/2}^{\left(i\right)})/{\left\{2(1+{\hat{\rho }}_{x,1/2}^{\left(i\right)})\right\}}^{1/2}$ for *x* = 0,1/2,1. Then, for CATT with a preselected genetic model, *P*_*x*_^(2)^ = 1 − Φ(sign(*Z*_*x*_^(1)^ · *Z*_*x*_^(2)^)·|*Z*_*x*_^(2)^|) using a one-sided *p* value given the direction of association from the discovery stage, and *P*_*x*_^(2)∗^ = 1 − Φ(sign(*Z*_*x*_^(1)∗^ · *Z*_*x*_^(2)∗^)·|*Z*_*x*_^(2)∗^|). Moreover, denote the test statistics and *p* values using Pearson's Chi-square test from the *i*th stage as *T*_chi2_^(*i*)^ and *P*_chi2_^(*i*)^. Further, let HWDTT from the *i*th stage be *Z*_*H*_^(*i*)^. Then, the second stage *p* values, using MAX3, MIN2, *Z*_GMS_, and *Z*_GME_, denoted as *P*_MAX3_^(2)^, *P*_MIN2_^(2)^, *P*_GMS_^(2)^, and *P*_GME_^(2)^, can be obtained as follows:

(7) PMAX3(2)=Px∗(2), where  x∗=arg min⁡x∈0,1/2,1⁡Px(1),PMIN2(2)=P1/2(2)·IP1/21≤Pchi22+Pchi22·IP1/21>Pchi22,PGMS(2)=P0(2)I(ZH(1)>c)+P1(2)I(ZH(1)<−c) +P1/22IZH1≤c, if  Z1/21>0;=P02I(ZH1<−c)+P12IZH1>c +P1/22IZH1≤c, if  Z1/2(1)≤0,PGME(2)=P0(2)∗I(ZH(1)>c)+P1(2)∗I(ZH(1)<−c) +P1/22∗IZH1≤c, if  Z1/2(1)>0;=P0(2)∗I(ZH(1)<−c)+P1(2)∗I(ZH(1)>c) +P1/22∗IZH1≤c, if  Z1/2(1)≤0.

It is important to note that even though the direction of the test statistics and the selected genetic models are used to obtain the second stage *p* values, the *p* values from the two stages are independent under the null hypothesis. This is because, under the null hypothesis, the probability of *Z*_1/2_ being positive or negative is simply 1/2, and the probability of the selection of a certain genetic model is also a constant (*α*_*H*_ for the recessive and dominant models and 1 − 2*α*_*H*_ for the additive model).

### 2.4. Combined Test Using *p* Values and Its Statistical Significance

For a given robust test, we can consider the joint analysis by combining *p* values from the discovery and replication stages of GWAS. We consider using *p* values rather than the test statistics because test statistics can have complex forms and obtaining the distribution of the joint test can be difficult. On the other hand, calculating a *p* value for each data set might be relatively simple, and the distribution of *p* values under the null hypothesis of no association is easy to handle.

There are several methods for combining test statistics from two stages [22], and two most commonly used forms are based on Fisher's combination and a linear combination after inverse normal transformation [23]. Fisher's combination (FC) directly sums *p* values after −2log⁡ transformation; that is, *Z*_FC_ = −2*w*_1_log⁡(*P* ^(1)^) − 2*w*_2_log⁡(*P* ^(2)^), where *P* ^(*i*)^ is *p* value from *i* = 0 for discovery and *i* = 1 for replication stages using a given robust test. A specification of *w*_1_ = *w*_2_ = 1 gives the same weight for discovery and replication stages, and one can consider *w*_1_ = 2*π*_*s*_ and *w*_2_ = 2(1 − *π*_*s*_) where *π*_*s*_ = *N*_*D*_/(*N*_*D*_ + *N*_*R*_), and *N*_*D*_ and *N*_*R*_ are sample sizes of the discovery and replication data sets. A linear combination of two *P* values after taking the inverse of the standard normal cumulative distribution is given by $Z_{\text{LC}}=\{w_{1}\Phi^{-1}(1-P^{\left(1\right)}/2)+w_{2}\Phi^{-1}(1-P^{\left(2\right)})\}/\sqrt{w_{1}^{2}+w_{2}^{2}}$ with a natural choice of $w_{1}=\sqrt{\pi_{s}}$ and $w_{2}=\sqrt{1-\pi_{s}}$. Let the significance level of the discovery stage be *α*_*D*_, which means that markers with *P* ^(1)^ < *α*_*D*_ are selected and replicated in the replication stage. The *p* value of combined test can then be obtained as *p*_FC_ = *P*_*H*_0__(*P* ^(1)^ < *α*_*D*_, *Z*_FC_ > *z*_FC_) where the observed value of *Z*_FC_ is *z*_FC_. The *p*_FC_ are calculated as *e* ^−*z*_FC_/2^(1 + *z*_FC_/2 + log⁡*α*_*D*_) for equal weights where *z*_FC_ > −2log⁡*α*_*D*_ and (*w*_1_/(*w*_1_ − *w*_2_))*e* ^−*z*_FC_/2*w*_1_^ − (*w*_2_/(*w*_1_ − *w*_2_))*e* ^−*z*_FC_/2*w*_2_^ *α*_*d*_^−(*w*_1_−*w*_2_)/*w*_2_^ for unequal weights where *z*_FC_ > −2*w*_1_log⁡*α*_*D*_. Detailed derivations are described in the Appendix. Equivalently, for an overall type I error threshold for a single marker of *α*, one may obtain the threshold *C*_FC_ of *Z*_FC_ that satisfies *P*_*H*_0__(*P* ^(1)^ < *α*_*D*_, *Z*_FC_ > *C*_FC_) ≤ *α*. Similarly, for the *Z*_LC_, the *p* value is calculated as *p*_LC_ = *P*_*H*_0__(*P* ^(1)^ < *α*_*D*_, *Z*_LC_ > *z*_LC_) = $\int_{z_{1-\alpha_{D}/2}}^{\infty }\phi (z)[1-\Phi ((\sqrt{w_{1}^{2}+w_{2}^{2}}z_{\text{LC}}-w_{1}z)/w_{2})]dz$ for *z*_LC_ > *z*_1−*α*_*D*_/2_ where the observed value of *Z*_LC_ = *z*_LC_.

## 3. Simulation Results

### 3.1. Type I Error

Table 1 provides the type I errors under different scenarios. A disease prevalence of 10% is assumed, and a total of 1500 cases and 1500 controls were divided into two stages. The proportions of samples in the first stage (*π*_*s*_) of 0.5 and 0.6 were considered for the minor allele frequency (MAF) of 0.3 and 0.4. We considered *M* = 10 markers to control the genome-wide false positive rate at *α* = 0.05 with the Bonferroni correction. We did not consider a larger *M* such as 300,000 or 500,000 because this will require more than 10 million simulations to show a stable estimate of the type I error rate. With *M* = 10, we performed 20,000 simulations which result in less than 10% of a coefficient of variation for a significance level 0.05/*M* = 0.005 for each marker [24]. The test statistics considered are *Z*_1/2_, Pearson's Chi-square test, MIN2, MAX3, GMS, and GME. For the second stage analysis, we considered a replication-based analysis, *Z*_FC_, and *Z*_LC_ as proposed above. The results are based on the situation under HWE (HWE coefficient *F* = 0). As expected, all tests control the type I error reasonablly well, and similar results were obtained when a slight deviation from HWE is present with *F* = 0.05 (results not shown).

### 3.2. Empirical Power

**Figure 1:**
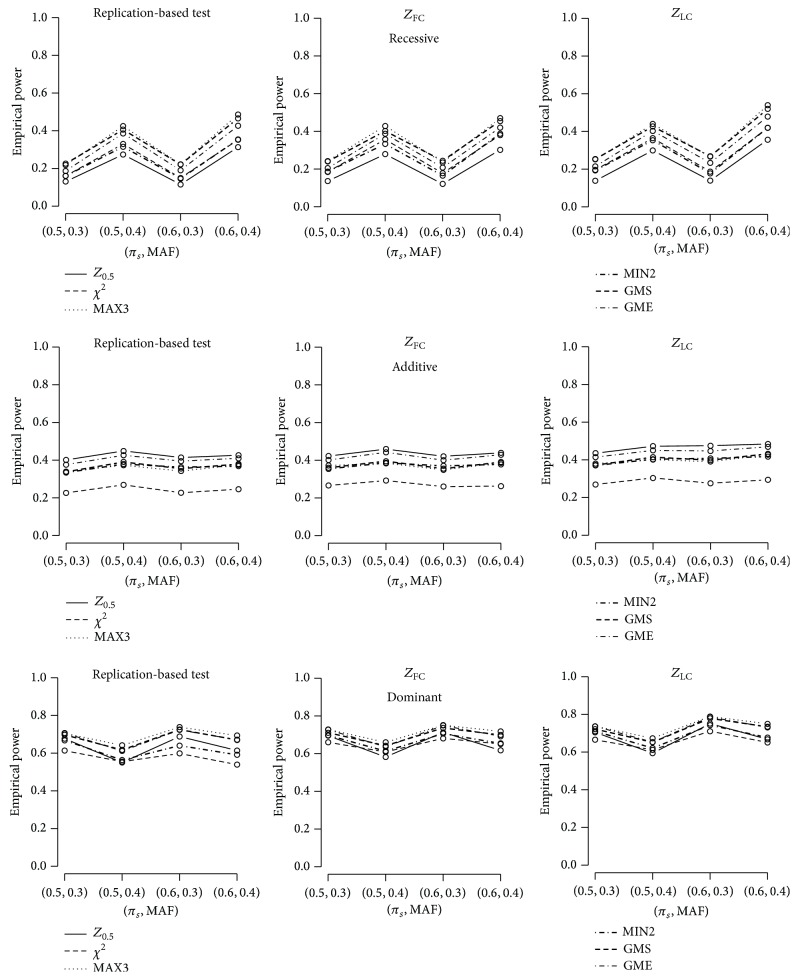
Empirical powers based on 2,000 simulations for *M* = 10 markers, genotype relative risks of both stages = 1.4, and disease prevalence *K* = 0.1 under the recessive, additive, and dominant models. 1,000 cases and 1,000 controls are considered to control *α* = 0.05. The first stage type I error rate for discovery is *α*_*D*_ = 0.05. Six test statistics, *Z*_1/2_, *χ* ^2^, MAX3, MIN2, GMS, and GME, are considered. The first, second, and third columns depict powers using the replication-based test, *Z*_FC_, and *Z*_LC_, respectively.

**Figure 2:**
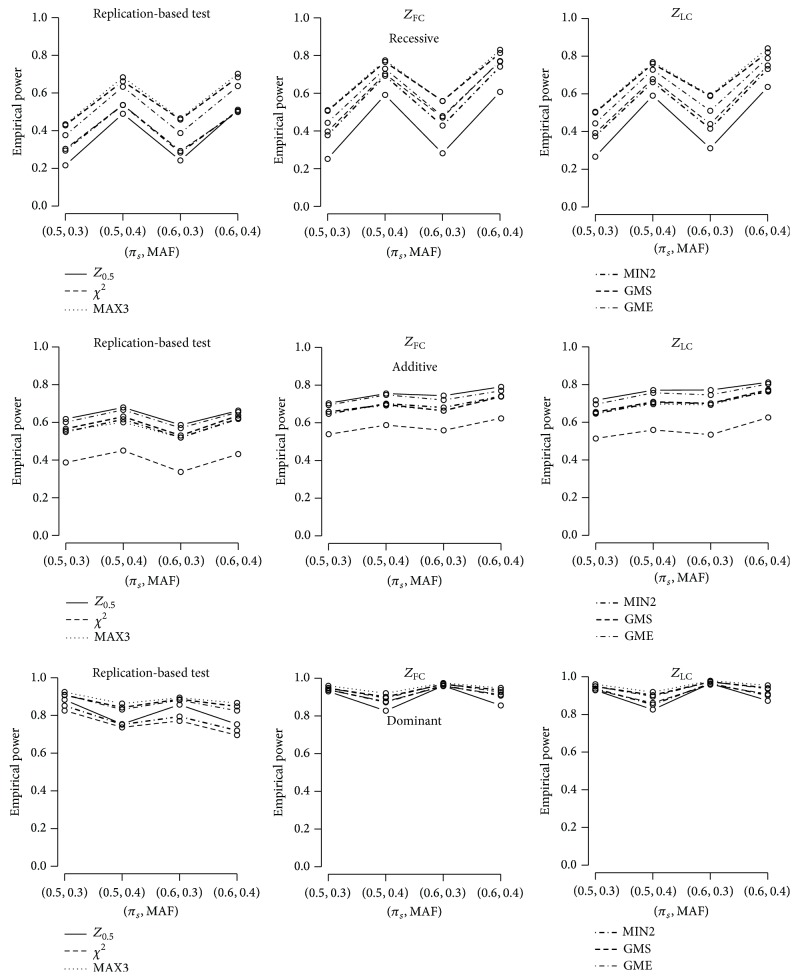
Empirical powers based on 2,000 simulations for *M* = 10 markers; genotype relative risks of two stages are different (*r*_1_ = 1.6, *r*_2_ = 1.4); disease prevalence *K* = 0.1 under the recessive, additive, and dominant models. 1,000 cases and 1,000 controls are considered to control *α* = 0.05. The first stage type I error rate for discovery is *α*_*D*_ = 0.05. Six test statistics, *Z*_1/2_, *χ* ^2^, MAX3, MIN2, GMS, and GME, are considered. The first, second, and third columns depict powers using the replication-based test, *Z*_FC_, and *Z*_LC_, respectively.

We examined the empirical powers of different tests considered above. In Figure 1, we considered *M* = 10 markers, a disease prevalence of 10%, the same genotype relative risk for two stages (*r*_1_ = 1.4 and *r*_2_ = 1.4), and 1,000 cases and 1,000 controls. 2,000 simulations were performed under HWE (*F* = 0) to control the genome-wide false positive rate at *α* = 0.05. The recessive, additive, and dominant models were assumed for the first, second, and third rows. Both joint analyses showed better power performances compared to the replication-based analysis (up to 15.9% in scenarios considered in Figure 1), and LC and FC have comparable powers with less than 2% difference. The power gain of using the joint analysis is not as much as that observed in Skol et al. [18]. However, as reported by Skol et al. [18], when the between-stage heterogeneity exists and the risk allele has a larger effect in the first stage than that in the second stage, much improved power is observed by using the joint test.  Figure 2 shows results under this scenario with *r*_1_ = 1.6 and *r*_2_ = 1.4, and the observed increase in power using the joint test is as high as 33.9%. Again, the difference between LC and FC is minor with less than 3% difference. As for comparison between different robust methods, MAX3, GMS, and GME perform well under the recessive model, while *Z*_1/2_, *χ* ^2^, and MIN2 are less powerful. Under the additive model, *Z*_1/2_ is most powerful, as expected, and *χ* ^2^ is least powerful. Other robust methods perform well with a slight decrease in power compared to *Z*_1/2_. Under the dominant model, MAX3, GMS, and GME perform the best even though all tests show good power performances, and the difference is minor. Similar patterns were observed when a slight deviation from the HWE is present (results not shown).

## 4. Real Data Application

**Figure 3:**
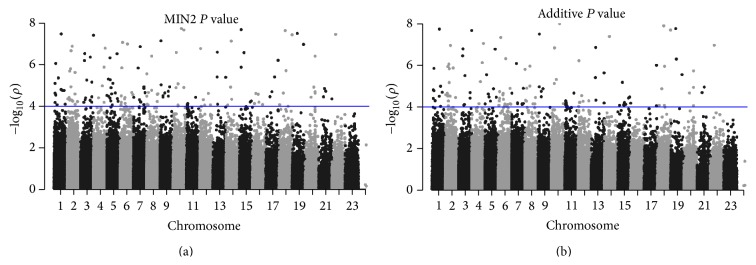
Manhattan plots of 246,758 SNPs from Yoon et al. [25] based on MIN2 (a) and *Z*_1/2_ (b). The *x* axis is chromosomal location and the *y* axis is the significance (−log⁡_10_ *P*) of association. The horizontal line corresponds to the significance level 10^−4^.

The GWAS on non-small-cell lung cancer (NSCLC) by Yoon et al. [25] studied 621 NSCLC patients and 1541 control subjects in the discovery stage. After stringent quality control steps, a total of 246,758 SNPs were tested for the association with NSCLC based on *Z*_1/2_. In the replication stage, 168 SNPs with *p* value less than 1 × 10^−4^ in the first stage based on *Z*_1/2_ were tested using 804 patients and 1470 control samples. We identified additional 234 SNPs using MIN2 in the first stage which could be studied in the replication stage if MIN2 was used instead of *Z*_1/2_ since MIN2 produces stronger evidence for the additional SNPs than *Z*_1/2_ does. The Manhattan plots of using MIN2 and *Z*_1/2_ are presented in Figure 3. One example is *rs*385272 located in chromosome 2, which had a *p* value of 1.37 × 10^−7^ which reached significance level at Bonferroni correction in discovery samples alone, whereas *Z*_1/2_ yielded a *p* value greater than 1 × 10^−4^. Even though there is possibility of false positive findings, these SNPs could have been selected for replication if robust methods were used.

Since we do not have replication data for these additional SNPs selected using MIN2 because the first stage selection was based on *Z*_1/2_ in Yoon et al. [25], just for illustration purpose of the proposed methods, we present the results of three SNPs including *rs*2131877 that was reported by Yoon et al. [25]. When the significance level in the discovery stage is set at *α*_*D*_ = 5 × 10^−5^ so that all these exemplary SNPs can be selected in the discovery stage; the *p* value of combined test based on four robust methods (MAX3, MIN2, GMS, and GME) as well as *Z*_1/2_ and Pearson's Chi-square test is presented in Table 2. Fisher's combination was used for the joint test in the second stage. Only *rs*2131877 was found to be significant with Bonferroni correction (*p* value <2.03 × 10^−7^) by all except MAX3 method.

## 5. Discussion

In genetic association studies, efficiency robust tests whose performance does not depend on the underlying genetic model have been extensively studied, and their power benefit over a wide range of genetic models has been well recognized. In this paper, we described how the idea of these robust association tests can be applied to the replication studies and further how overall statistical significance can be evaluated using the combined test formed by *p* values of the discovery and replication studies.

When the robust tests are used, the test statistic of each stage can have a complex form and thus dealing with the distribution of the joint test can be difficult, whereas calculating the *p* value of each stage might be relatively simple. Because the asymptotic distribution of the *p* value under the null hypothesis of no association is easy to handle, the combined test using *p* values rather than the test statistics themselves can provide computational convenience.

There are several methods for combining test statistics from two stages and Won et al. [22] compared the performances of various choices. Two most commonly used forms are based on Fisher's combination and the linear combination after the inverse normal transformation [23], and we presented the test statistics and *p* values of these two methods. In our limited experience, the linear combination and Fisher's combination are fairly comparable. Fisher's combination seems to perform slightly better than the linear combination when there exists some heterogeneity between stages in terms of the genotype relative risk, while the linear combination seems to perform slightly better in most of other situations. However, the difference is extremely minor. Further research is required for the thorough comparison of various methods of combining *p* values in the application of efficiency robust tests to the replication of genetic association studies.

In a genetic study where the purpose of considering a replication stage is to validate or replicate the genetic findings from the discovery stage, which is the case considered in this paper, the analysis in the replication stage utilized the test statistic or genetic model that is selected as being the best in the discovery stage and also the direction of the risk allele, following guidelines for exact replication in genetic association studies. If the purpose is to simply combine the evidence from different data sources such as in meta-analysis, other strategies may be devised. Further research, again, is required to provide fully detailed properties of such methods.

Power gain of a joint analysis over the conventional replication-based analysis was thoroughly studied by Skol et al. [18, 19]. In our simulation, the amount of power increase using a joint test compared to the replication-based analysis was much minor than what was observed by Skol et al. [18, 19]. The exact reason is not known, but we suspect this might be due to the power advantages of robust methods and also due to the fact that the optimal choice from the first stage is used when calculating the second stage *p* values. However, even though it was minor in some situations, the joint anlysis presented better power performance than the replication-based analysis in our study. This type of joint analysis raised concerns about the exact meaning of replication [17]. However, McCarthy et al. [26] mentioned that joint analyses “blur the boundaries of where exactly replication starts, but whichever analytical approach is taken, confirmation in many independent samples is important and it is the overall strength of the evidence of association that matters.” Purpose of the current study was to present how the overall strength of the evidence of association can be evaluated when robust tests are used in GWAS replication studies.

We illustrated how the proposed methods can be applied in the real data that studied the association of SNPs with non-small-cell lung cancer (NSCLC) in discovery and replication stages. In the original study reported by Yoon et al. [25], SNPs were selected in the discovery data set not based on the robust tests but based on additive CATT. Therefore, we found that some SNPs could have been selected by one of the robust methods but they were not included in the replication data set. For these SNPs, we were not able to perform the joint analysis that we propose, and it was not possible to examine whether there are other SNPs that could have been found to be associated with NSCLC by proposed methods in the replication study. For this reason, we merely presented how many additional SNPs could have been further followed in the replication stage when robust methods were used. In many GWASs, it is a common practice to report the summary test statistics and *p* values of the SNPs under a specific genetic model, usually an additive model, which were further genotyped in the replication stage and were finally defined to be significantly associated with a phenotype of interest. As emphasized in this paper, one may have a better chance of finding many missing SNPs by applying more powerful and robust methods that consider different genetic models simultaneously. Therefore, we urge the community to share test results under not only an additive model but also other genetic models, although they were not significant at a stringent significance level, so that future research may have enriched data resources, to which robust tests can be applied in association studies.

## Figures and Tables

**Table 1:** Type I error rates of three approaches—replication-based (REP) test, Fisher's combination (*Z*_FC_), and linear combination of test (*Z*_LC_)—based on the CATT with an additive model (*Z*_1/2_), *χ* ^2^, MAX3, MIN2, GMS, and GME. The disease prevalence *K* = 0.1, *M* = 10 markers, *r* = 1,500 cases, and *s* = 1,500 controls are considered based on 20,000 simulations.

MAF	*π*_*s*_	*α*_*D*_		*F* = 0					
				*Z*_1/2_	*χ* ^2^	MAX3	MIN2	GMS	GME
0.3	0.5	0.05	REP	0.00530	0.00455	0.00505	0.00485	0.0050	0.00490
			*Z*_FC_	0.00500	0.00535	0.00495	0.00510	0.00515	0.00460
			*Z*_LC_	0.00535	0.00525	0.00485	0.00510	0.0050	0.00485
		0.1	REP	0.00510	0.00560	0.00565	0.00485	0.00525	0.00545
			*Z*_FC_	0.00565	0.00535	0.00565	0.00545	0.00565	0.00540
			*Z*_LC_	0.00520	0.00565	0.00525	0.00520	0.00530	0.00525

0.3	0.6	0.05	REP	0.00510	0.00485	0.00480	0.00515	0.00480	0.00500
			*Z*_FC_	0.00445	0.00455	0.00450	0.00455	0.00450	0.00460
			LC	0.00500	0.00515	0.00495	0.00520	0.00475	0.00480
		0.1	REP	0.00500	0.00485	0.00485	0.00535	0.00530	0.00505
			*Z*_FC_	0.00465	0.00490	0.00485	0.00500	0.00455	0.00460
			*Z*_LC_	0.00480	0.00470	0.00515	0.00490	0.00485	0.00475

0.4	0.5	0.05	REP	0.00590	0.00505	0.00530	0.00565	0.00505	0.00510
			*Z*_FC_	0.00575	0.00430	0.00460	0.00535	0.00460	0.00500
			*Z*_LC_	0.00600	0.00445	0.00500	0.00540	0.00490	0.00490
		0.1	REP	0.00525	0.00470	0.00535	0.00450	0.00480	0.00515
			*Z*_FC_	0.00515	0.00510	0.00495	0.00475	0.00540	0.00500
			*Z*_LC_	0.00530	0.00500	0.00485	0.00475	0.00495	0.00510

0.4	0.6	0.05	REP	0.00475	0.00585	0.00480	0.00500	0.00515	0.00495
			*Z*_FC_	0.00460	0.00470	0.00420	0.00490	0.00455	0.00440
			*Z*_LC_	0.00525	0.00550	0.00520	0.00580	0.00510	0.00510
		0.1	REP	0.00550	0.00490	0.00515	0.00535	0.00555	0.00540
			*Z*_FC_	0.00520	0.00370	0.00495	0.00450	0.00515	0.00510
			*Z*_LC_	0.00565	0.00485	0.00570	0.00530	0.00610	0.00580

**Table 2:** For selected exemplary three SNPs for testing association with NSCLC, *p* value of combined test using additive CATT (*Z*_1/2_), Pearson's Chi-square test (*T*_chi2_), MAX3, MIN2, *Z*_GMS_, and *Z*_GME_.

SNP	*p* value of *Z*_1/2_			*p* value of *T*_chi2_		
	Discovery	Replication	Combined test	Discovery	Replication	Combined test
rs2131877	7.88 × 10^−5^	1.04 × 10^−4^	7.97 × 10^−8^	1.40 × 10^−4^	1.49 × 10^−4^	1.84 × 10^−7^
rs905551	1.83 × 10^−5^	7.02 × 10^−3^	7.70 × 10^−6^	8.06 × 10^−5^	4.89 × 10^−2^	1.40 × 10^−5^
rs1695109	2.48 × 10^−4^	3.46 × 10^−2^	2.17 × 10^−6^	4.56 × 10^−5^	1.53 × 10^−1^	2.07 × 10^−5^

SNP	*p* value of MAX3			*p* value of MIN2		
	Discovery	Replication	Combined test	Discovery	Replication	Combined test

rs2131877	1.53 × 10^−4^	4.05 × 10^−2^	1.92 × 10^−5^	1.32 × 10^−4^	1.04 × 10^−4^	1.26 × 10^−7^
rs905551	4.50 × 10^−5^	7.02 × 10^−3^	1.92 × 10^−6^	1.34 × 10^−4^	4.89 × 10^−2^	1.99 × 10^−5^
rs1695109	3.54 × 10^−5^	2.63 × 10^−2^	4.64 × 10^−6^	2.36 × 10^−5^	2.63 × 10^−2^	3.35 × 10^−6^

SNP	*p* value of *Z*_GMS_			*p* value of *Z*_GME_		
	Discovery	Replication	Combined test	Discovery	Replication	Combined test

rs2131877	1.86 × 10^−4^	1.04 × 10^−4^	1.71 × 10^−7^	1.03 × 10^−4^	1.04 × 10^−4^	1.02 × 10^−7^
rs905551	5.19 × 10^−5^	7.02 × 10^−3^	2.16 × 10^−6^	7.35 × 10^−5^	8.01 × 10^−3^	3.20 × 10^−6^
rs1695109	6.89 × 10^−4^	1.27 × 10^−1^	3.85 × 10^−5^	2.69 × 10^−5^	4.19 × 10^−2^	5.40 × 10^−6^

## Appendix

### *p* value of Fisher's Combination for Equal and Unequal Weights

*Equal Weights*. Assume *w*_1_ = *w*_2_ = 1. Under the null hypothesis of no association, *X*_1_ = −2log⁡*P* ^(1)^ and *X*_2_ = −2log⁡*P* ^(2)^ are independent and each asymptotically follows a *χ* ^2^ distribution with 2 degrees of freedom Let *f*_*k*_(*x*) and *F*_*k*_(*x*) be the probability and cumulative density functions of *χ* ^2^ random variable with *k* degrees of freedom Then *f*_2_(*x*) = exp⁡(−*x*/2)/2, *f*_4_(*x*) = *x*exp⁡(−*x*/2)/4, *F*_2_(*x*) = 1 − exp⁡(−*x*/2), and *F*_4_(*x*) = 1 − exp⁡(−*x*/2) − *x*exp⁡(−*x*/2)/2. Denote the cutoff of the discovery stage based on *α*_*D*_ as *C*_*D*_; that is, *F*_2_(*C*_*D*_) = 1 − *α*_*D*_. For observed value *z*_FC_ > *C*_*D*_ of *X*_1_ + *X*_2_, the *p* value is written as

(A.1) PH0X1>CD,X1+X2>zFC =αD−∫CDzFCf2xF2zFC−xdx =αD+exp⁡−zFC2−αD+12exp⁡−zFC2(zFC−CD) =exp⁡−zFC21+zFC2+log⁡αD.

*Unequal Weights.* When different proportions of samples are used in the discovery and replication stages, it may be more appropriate to assign weights proportional to the sample sizes for each stage. For example, when only a small portion is used in the discovery stage, to prevent Fisher's combination test from being dominated by the significant result in the discovery stage, one may want to assign a small weight to the discovery stage result.

When *π*_*s*_ is the proportion of samples used in the discovery stage, one selection for weights is *w*_1_ = 2*π*_*s*_ and *w*_2_ = 2(1 − *π*_*s*_) for discovery and replication stages, which simplifies to equal weights when *π*_*s*_ = 0.5. Based on these weights, we consider unequal-weighted Fisher's combination as −2log⁡*P* ^(1)*w*_1_^ *P* ^(2)*w*_2_^ = *w*_1_ *X*_1_ + *w*_2_ *X*_2_ [27]. Its density function is given by

(A.2) fwx=12w1−w2exp⁡−x2w1−12w1−w2exp⁡−x2w2,

and the probability distribution function is

(A.3) Fwx=1−w1w1−w2exp⁡−x2w1   −w2w1−w2exp⁡−x2w2, w1≠w2.

Using the previous notation, we have the following form of *p* value:

(A.4) PH0X1>CD,w1X1+w2X2>zFC =αD−∫CDzFC/w1f2(x)F2zFC−w1xw2dx =exp⁡−zFC2w1+w2w1−w2exp⁡−zFC2w2  ×exp⁡w1−w22w1w2zFC−exp⁡w1−w22w1w2w1CD =w1w1−w2exp⁡−zFC2w1  −w2w1−w2exp⁡−zFC2w2αD−w1−w2/w2 =w1w1−w2exp⁡−zFC2w1  −w2w1−w2exp⁡−zFC2w2αD−(w1−w2)/w2,

where *z*_FC_/*w*_1_ > *C*_*D*_.
